# Psychometric properties of the caregiving difficulty scale in mothers of children with cerebral palsy

**DOI:** 10.1186/s12883-023-03264-w

**Published:** 2023-06-20

**Authors:** Eun-Young Park

**Affiliations:** Department of Secondary Special Education, Jeonju University, Jeonju, 55069 South Korea

**Keywords:** Mothers, Children, Cerebral palsy, Rasch, Psychometric

## Abstract

**Background:**

The Caregiving Difficulty Scale is used to measure the burden of caregiving experienced by mothers of children with cerebral palsy. This study aimed to identify the psychometric properties of the Caregiving Difficulty Scale using the Rasch model.

**Methods:**

Data collected from 206 mothers of children with cerebral palsy were analyzed. Unidimensionality, difficulty of item, rating scale appropriateness, and reliability using the separation index of the Caregiving Difficulty Scale were verified. Unidimensionality of all 25 items was identified through the item fit.

**Results:**

Our analysis of item difficulty indicated that person ability and item difficulty are expressed as a similar logit extend. The use of the 5-point rating scale appeared to be appropriate. Outcome analysis revealed that the reliability was high based on the person and that the item separation level was acceptable.

**Conclusions:**

This study showed that the Caregiving Difficulty Scale could be a valuable tool for evaluating the caregiving burden in mothers of children with cerebral palsy.

## Background

Cerebral palsy (CP) is a collective term encompassing etiologically diverse symptoms. CP is a group of disorders of motor function, movement, and postural development that results in limited activity due to a non-progressive disorder in a developing fetus or infant [1]. Motor function disorders are core symptoms of CP and are frequently accompanied by other dysfunctions such as sensory, cognition, communication, and perception [1]. Furthermore, CP has many other commodities such as spasticity, contractures, dystonia, poor balance, abnormal bone growth, loss of selective motor control, and significant limitation in daily activities such as mobility, self-care, and social functioning [2]. CP is the most common childhood chronic disease [3], with a prevalence rate of 2.08 per 1000 live births [4]. The cerebral palsy community presents high dependency and low occupational performance, which leads to the need for a third person to perform or support activities of daily living.

Most children with disabilities have remained at home under family protection rather than at an institution. Children with long-term functional limitations require care at home; however, it can impact the health and quality of life of the caregivers [5, 6]. Caring for a child with a disability comes with many challenges and stresses [5, 7, 8]. Mothers of children with CP have higher scores on stressful life events, a lower sense of self-mastery, a limited network of friends, a lower level of mental health, and more difficulties in marital adaptation than mothers of children without physical disabilities [5]. The families of children with disabilities have a poor quality of life due to the socioeconomic burden, reduced social relations, and psychological departure associated with caring for and educating their children with CP [8]. However, due to various reasons, the available service, support, or policies are still insufficient for problematic situations faced by children with CP and their families, and the protection of the children is entirely performed at the family level.

Among the stress-inducing factors facing these families, the caregiving burden is reportedly the most influential factor in parental well-being [8, 9]. The parenting burden leads to psychological changes such as depression, manifestation of depression-related symptoms, insomnia, and lack of motivation [10–12]. Caregiving burden has been reported to decrease subjective psychological well-being, physical health, and life satisfaction in caregivers [9, 13]. Piran et al. [14] reported a moderate level of caregiving burden among 249 caregivers of children with chronic diseases and a maximum burden among caregivers of children with CP. Brehaut et al. [15] stated that the reports of chronic physical and mental illness, activity limitations, and elevated depressive symptoms in caregivers of children with health problems were more than double than in caregivers of healthy children, alongside a higher rate of poorer general health.

The unaddressed burden of caring for children with CP will inevitably negatively impact the lives of the children, their families, and especially their caregivers. Therefore, it is important to identify factors related to the burden of care, find ways to alleviate this burden, and accurately measure the degree of caregiving burden [16–18]. The primary caregiver of a child with disabilities is the mother, and this is also the case for children with CP [19]. Accurately measuring the caregiving burden experienced by the mothers of children with CP is the first step towards improving the mental health of the mothers and supporting their families. Several tools such as the Burden Assessment Schedule, Caregiver Burden Inventory, and Zarit Burden Scale have been developed to assess the burden of care. Although some of these tools are available to measure the burden of care required by children with CP, they did not examine psychometric properties in these children. A tool developed within the past decade is the Caregiver Difficulty Scale (CDS) [16]. However, since the CDS has been developed relatively recently and is yet to be widely used in clinical settings, there is a need for an alternative to the CDS and studies to confirm its psychometric properties. Wijesinghe et al. [16] reported the multidimensionality of the CDS that includes the caregiver’s concerns for the child, effect on self, support received for caregiving, social and economic strain, the validity of the construct, content, face, content validity, and internal consistency. Although these authors [16] reported that the level of internal consistency was satisfactory, subscale 3 (support for caregiving) of the CDS was 0.689, which was not acceptable. Farajzadeh et al. [3] evaluated the construct validity and reliability of the Persian version of the CDS and reported that the fit indices were adequate and that the Tucker–Lewis index was 0.88. Park [20] reported a bi-factor model comprising four sub-factors.

Recently, attempts have been made to verify the items derived from the confirmatory factor analysis with different statistical methods to evaluate the fit and difficulty of the items more accurately [21, 22]. The item response theory is a method of analyzing items by their unique, individual curves. The difficulty and discrimination of each question can be analyzed, and the actual ability of the participant can be estimated based on the analysis result. The item characteristic estimation is advantageous since it is unaffected by the features of the participant group [23, 24]. However, item analysis in the CDS, a tool developed to measure the burden of care for children with CP, has not yet been reported. In order to further accurately measure the caregiving burden for children with CP, it is necessary to use a tool developed for children with CP. In this regard, the CDS will be translated and its psychometric characteristics will be verified. In the case of a translated tool, it is essential to determine and validate the psychometric properties.

Therefore, this study aimed to examine the psychometric characteristics of the CDS using Rasch analysis for caregiving difficulty in mothers of children with CP. Specifically, the study examined the following: (a) the appropriateness of the CDS items in assessing the caregiving burden for mothers of children with CP, (b) item difficulty, (c) reliability based on the separation index, and (d) the appropriateness of the CDS rating scale.

## Methods

### Data

This study was approved by the Research Ethics Board of Jeonju University (Jeonju University IRB-1041042-2013-1). Children with CP were either involved in community welfare centers or undergoing rehabilitation at hospitals or medical centers. Before the survey, the researcher sent letters to colleagues in involved institutes regarding referrals of eligible mothers of children with CP. Once the possible number of mothers was identified, they were informed about the research process, and written consent was obtained for participation in this study. In total, 215 mothers of children with CP participated in this study. For data collection, the mothers received self-report questionnaire on CDS, and data on general characteristics, such as age, education level, and employment status were collected. The Gross Motor Function Classification System (GMFCS) data on level and type of motor function impediment were obtained from the physical therapists of children with CP. The detailed explanation about CDS response method was provided to mothers, and they were encouraged to address any question or problem to the researcher during response. The inclusion criterion was a diagnosis of CP, and there were no specific exclusion criteria, except the age range, as children were required to be 3–15 years of age to be included in the study. Among the 215 participants, nine with inadequate data for the Rasch analysis were excluded because their standard infit mean square (MNSQ) exceeded 2.0. Since a well-targeted sample of 50 responses is required to obtain a useful and reliable estimate [25], the sample size of this study was sufficient for analysis.

The mean age of the children with CP was 8.5 years (SD = 3.5). There were 129 boys (62.6%) and 77 girls (37.4). According to the GMFCS, 11.2%, 8.7%, 6.8%, 16.5%, and 56.8% of the children were classified as Level 1 (n = 23), Level 2 (n = 18), Level 3 (n = 14), Level 4 (n = 34), and Level 5 (n = 117), respectively. Moreover, 77.2%, 13.6%, and 9.2% of the children had spastic (n = 159), dyskinetic (n = 28), or ataxic (n = 19) CP, respectively. In addition, 35.4%, 60.2%, and 4.4% of the mothers were 30–39 (n = 73,), 40–49 (n = 124,), and ≥ 50 (n = 9) years old, respectively. In terms of education, 77.2% (n = 159) of the mothers were college graduates, 20.9% (n = 43, 20.9%) were high school graduates, and 0.5% (n = 1) were middle school graduates. Fifty-nine mothers (28.6%) were employed, and 154 (69.9%0) were unemployed. The characteristics of the participants are presented in Table 1.

**Table 1:** General characteristics of participants

Category	Sub-category		n	%
Children	Sex	Boy	129	62.6
		Girl	77	37.4
	Type	Spastic	159	77.2
		Dyskinetic	28	13.6
		Ataxic	19	9.2
	GMFCS	Level 1	23	11.2
		Level 2	18	8.7
		Level 3	14	6.8
		Level 4	34	16.5
		Level 5	117	56.8
Mothers	Age	30 ~ 39	73	35.4
		40 ~ 49	124	60.2
		50 ≤	9	4.4
	Education Level	College graduate	159	77.2
		High school graduate	43	20.9
		Middle school graduate	1	0.5
		Missing value	3	1.5
	Employment	Yes	59	28.6
		No	144	69.9
		Missing value	3	1.5

Note: GMFCS = Gross Motor Function Classification System

### Measure

#### Gross motor function classification system

The GMFCS was used to evaluate the gross motor function of children with CP. It is a tool developed to evaluate movement disorders in children with CP in five levels: level 1, the patient can walk with no restrictions; level 2, the patient can walk with limited mobility; level 3, the patient can walk without trunk support, canes, crutches, or walkers; level 4, the movement is limited, but an electric wheelchair can be used, the patient can move independently using other means of transport; level 5, the patient has severely limited mobility even with assistive devices [26].

#### CDS

The CDS is a practical tool to test the caregiving burden among caregivers of children with CP. It was developed by Wijesinghe et al. [16]. Park [20] translated the CDS into Korean and verified the validity of the Korean version (K-CDS). The CDS consists of four subscales that measure eight items about the Concern from the Child subscale, seven items about Impact on Self, five items about Support for Caregiving, and five items about Social and Economic Strain. There are 25 items measured on the 5-point scale, ranging from 0 to 4. The total score ranges from 0 to 100. Cronbach’s alpha of the CDS was reported to be 0.892 [20].

### Statistical analysis

Statistical data were analyzed using the software WINSTEPS 3.6 [27] by applying the Rasch model. Rasch analysis based on item response theory is a method of analyzing items according to item characteristic curves unique to each item. It has the advantage of providing the ability to analyze the difficulty and discrimination of each item, estimation of the person’s true ability based on the analysis results. Further, the characteristics of the responder does not impact the results of this method [28].

#### Unidimensionality

When the MNSQ value of items was not suitable, they were judged as a misfit. The criteria were infit MNSQ values < 0.5 or > 1.5 and z-values <-2.0 or > 2.0 [27]. Each infit and outfit MNSQ of 1.0 was considered the ideal value in the Rasch model, which means that the data showed a perfect fit [28]. Items with MNSQ values > 1.3 indicate no construct heterogeneity with other items on the scale, while values < 0.7 indicate item redundancy with other items. Z-values are used to determine misfits with MNSQ value; z-values <-2.0 or > 2.0 indicate an inadequate fit for each item. Since infit MNSQ is influenced by the response patterns and is generally difficult to diagnose and remedy, infit MNSQ poses a greater threat to the measurement; since outfit MNSQ values are influenced by outliers, and are easy to diagnose and correct, infit MNSQ values were used in this study [29].

#### Item difficulty

The Rasch model Wright map was used to examine the item difficulty of the CDS. The Rasch model Wright map shows persons and items, and the distribution of respondents in the sample on a map. Rasch analysis presents both a person’s ability and item difficulty by converting them into logit values, expressed as the natural logarithm of the probability that a person can perform a specific task. The probability that a person can perform a particular task is the ratio of the probability of being able to perform the task to the probability that the task cannot be performed. A larger positive-sized logit indicates an increased item difficulty [29]. Direct comparison is possible by converting individual attribute scores and item difficulty into the same logit scale, making it possible to evaluate whether item difficulty is suitable for the analysis of the target group. A distribution can be said to be appropriate when the ranges of the two distributions match, that is when the range of distributions is similar enough that the item difficulty can measure all ranges of individual attributes [30].

#### Reliability

Reliability was determined using separation reliability statistics. In the Rasch analysis, person separation was done using Cronbach alpha [31]. The separation index indicates the number of different strata of responders that can be segregated by measures of caregiving difficulties [32]. To achieve the desired confidence level of at least 0.80, the segregation index must be greater than 2 and must exceed at least 3 to achieve a confidence level of 0.90 [29].

#### Rating scale analysis

Categorical functions were analyzed within the Rasch model to examine the suitability of the CDS rating scale (5-point scale). Since the respondents did not respond to the rating scale in the intended way and were uncertain about how to use the response options, we assessed the suitability of the rating scale. The fitted values for each rating also provide information on whether each rating works well. Since a value of 1.0 is considered an ideal value, a fitted value of > 1.5 in an individual response category indicates the ineffective functioning of the rating scale [27, 31]. A threshold that does not continuously increase or decrease is judged to be inconsistent with the level of the construct at which the response to the item is measured.

## Results

### Unidimensionality

The results of the CDS item appropriateness analysis are summarized in Table 2. The results of the item fit test judged one item to be a misfit: Item 20 (Do your relatives/neighbors help you with caring for the child?) had an infit value of 1.62 and a z-value of 5.7.

**Table 2:** Item fit statistics

Item no.	Measure	SE	Infit		Outfit	
			MNSQ	Z-value	MNSQ	Z-value
1	54.89	0.71	0.89	-1.3	0.94	-0.6
2	51.42	0.71	0.99	-0.1	1.04	0.5
3	37.44	0.90	0.95	-0.5	0.88	-1.0
4	38.16	0.88	0.88	-1.1	0.86	-1.1
5	43.19	0.78	0.97	-0.3	0.94	-00.5
6	48.10	0.72	0.90	-1.1	0.89	-1.2
7	44.72	0.76	0.84	-1.7	0.87	-1.4
8	45.85	0.74	0.93	-0.8	0.92	− 0.8
9	48.42	0.72	0.59	-5.4	0.59	-5.3
10	49.76	0.71	0.83	-2.0	0.82	-2.1
11	44.78	0.76	0.98	-0.2	1.07	0.7
12	50.32	0.71	0.93	-0.8	0.95	-0.6
13	49.91	0.71	0.76	-2.9	0.76	-2.9
14	50.06	0.71	0.77	-2.6	0.78	-2.6
15	53.18	0.71	0.79	-2.4	0.80	-2.4
16	58.79	0.73	1.33	3.2	1.32	3.2
17	57.93	0.73	1.32	3.8	1.39	3.8
18	59.00	0.74	1.12	0.9	1.08	0.9
19	63.41	0.79	1.03	-0.3	0.97	-0.3
**20**	**49.45**	**0.71**	**1.62**	**5.7**	**1.62**	**5.7**
21	56.57	0.72	1.33	2.9	1.28	2.9
22	51.22	0.71	0.92	-1.0	0.91	-1.0
23	41.63	0.81	1.24	1.8	1.20	1.8
24	48.31	0.72	1.34	4.0	1.41	4.0
25	53.48	0.71	1.03	0.2	1.02	0.2

Note: SE = standard error, MNSQ = mean square

### Item difficulty

**Fig. 1:**
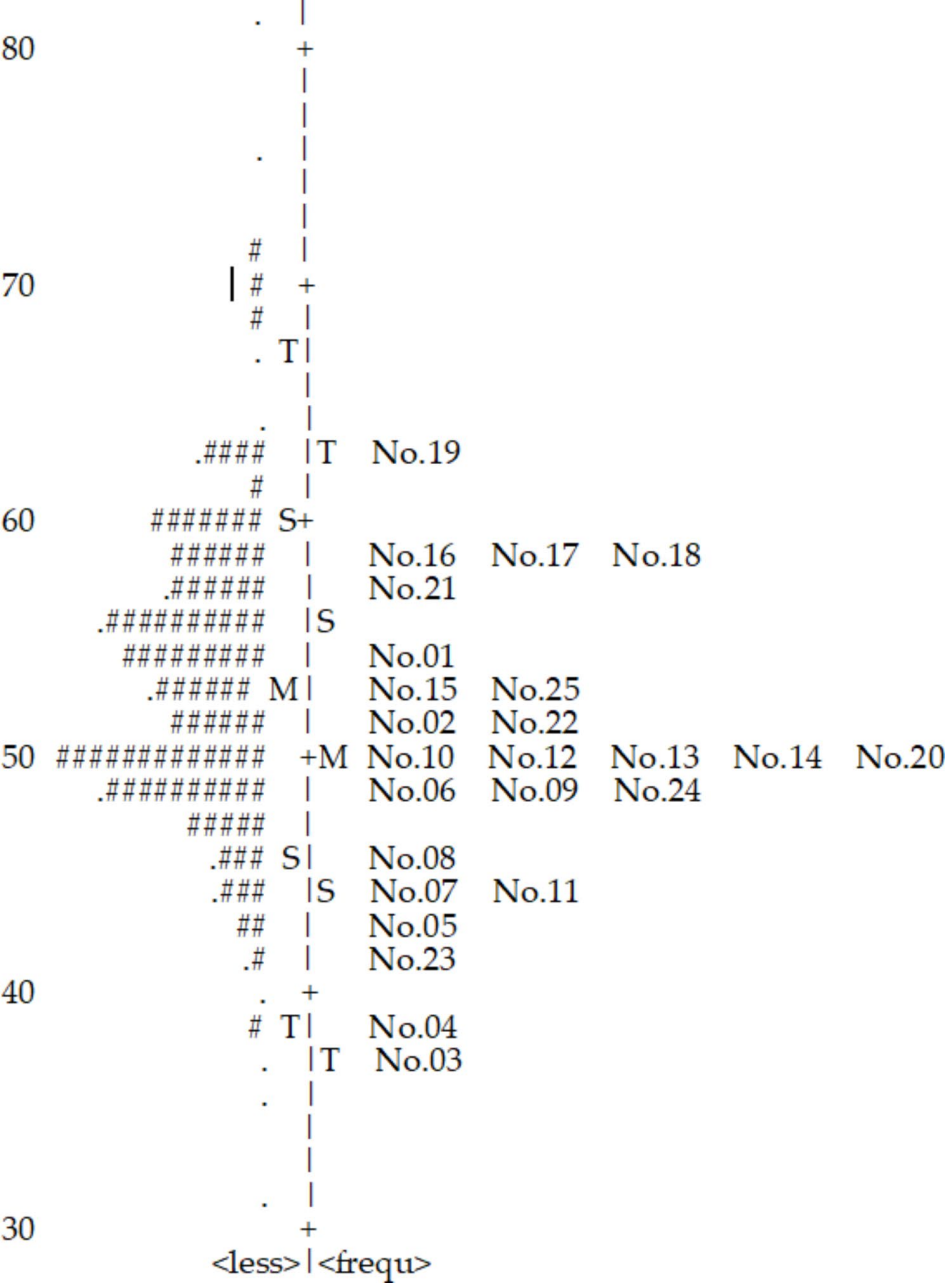
A map of an individual ability and item difficulty for the 25 items on Caregiving Difficulty Scale (CDS). Each (#) represents two mothers of children with CP; more, high personal ability; less, low personal ability; rare, high item difficulty; frequ, low item difficulty; +M, item mean; M, person mean; S = 1 SD from the mean; T = 2 SD from the mean. Mother’s location range was similar to the range of an item’s difficulty level

The results of the item difficulty analysis using the Wright maps showed that for this sample of mothers of children with CP, the CDS person mean and item means were similar, indicating appropriate difficulty levels (Fig. 1). Item 19 was the most difficult, and Item 3 was the easiest. Six mothers of children with CP showed higher ability estimates than that for the most difficult Item 19. No mothers of children with CP showed the abilities lower than the ones corresponding to the easiest Item 3.

### Reliability

The person separation index was 2.79, indicating an acceptable level of separation that functioned independently and distinctly. Cronbach’s alpha was 0.89, which was based on the person separation index. The item separation index was 3.06, indicating an excellent level of separation functioning independently and distinctly.

### Rating scale

The results of analyzing the 5-point rating scale format of the CDS using the item fit approach are shown in Table 3. This table shows that the average measures of the five categories increased monotonically and that the fit statistics confirmed that the category function was good because the infit and outfit MNSQs for all categories were < 1.5.

**Table 3:** Rating scale analysis of the Caregiving Difficulty Scale

Category	Observed Count	Average Measure	Infit MNSQ	Outfit MNSQ	Structure Measure
0	505	-6.88	1.13	1.14	None
1	926	-2.94	0.99	0.97	-11.29
2	1451	1.57	0.97	0.98	-5.04
3	1247	5.97	1.03	0.99	5.59
4	1071	12.75	0.95	0.97	10.74

Note: MNSQ = mean square

## Discussion

Caring for children with CP is a burden on the parents and their families [8]. There are limited studies that examine the caregiving burden on mothers of children with CP. The need for effective tools to measure the pain and burden of children and families is a fundamental premise for promoting integrated ICF-based rehabilitation and social strategies that promote the well-being of children and families. The tools described herein can be outcome measures for tracking the appropriateness of an intervention over time. A recent study reported that this caregiving burden is related to an increase in the chances of depression and a decrease in the quality of life of mothers of children with CP [33]. This study was conducted to investigate the psychometric characteristics of CDS, a tool developed to measure the burden of care for children with CP, using a Rasch analysis.

The first research question was whether items of the CDS are fit for unidimensionality. Most items, except item 20, produced a unidimensional attribute based on the results from the item fit statistics. This item showed that it was possibly unproductive for the construct of measurement. However, the infit MNSQ of this item was 1.62, which indicates that it is not degrading [34]. The exclusion of item 20 was not required; however, a revision was needed to indicate productivity for the construction of the measurement. The criteria for determining item suitability based on the MNSQ value are not absolute, and different criteria may be used depending on the purpose of the scale. When making a clinical observation, a relatively large fit MNSQ value criteria is used; specifically, a range of 0.5–1.7 is recommended for a reasonable infit and outfit MNSQ of the item [35].

The second research question addressed the item difficulty of the CDS. When the person mean is higher than the item mean, the items are relatively easy to endorse. Figure 1 showed that the person means of the CDS scale was higher than the item means. This implies that the items of CDS were easy for the mothers of children with CP. Easy items were those regarding #3 (Do you fear what our child’s future might be?) and #4 (Do you worry about your child’s present state?). Six mothers of children with CP had a higher ability than the item’s difficulty. Only 2% of the mothers of children with CP had an ability higher than that measured by the item. Although the person mean appeared to be higher than the item mean, the range between the range of item difficulty and the degree of endorsement of the item appeared similar. Finally, the item difficulty of CDS was at an acceptable level.

The third research question was an attempt to answer whether the rating scale of CDS was appropriate. Rasch analysis enables judgment by calculating and presenting the fitness values for individual response categories. The response scale used for measurement should have a clear response level and the possibility to measure the desired variable [36]. A fitness index of an individual response scale > 1.5 indicates that the response scale did not function properly, suggesting the formation of a new scale by combining the inappropriate response scale with other response scales [27]. Another criterion for determining whether an individual response scale is functioning adequately is to have at least 10 cases per rating category, with the mean measure monotonically increasing across each category, step calibration differences monotonically increasing, and determining whether the step calibration difference is > 1.4 [22]. The results showed that the 5-point response scale of the test was appropriate.

The fourth research question examined reliability, referring to the person and item separation indices. The person separation index is interpreted similarly to the same concept of Cronbach’s alpha [37]. Generally, it is suggested that a person separation reliability of 0.5 can classify 1 or 2 levels, 0.8 can differentiate 2 or 3 levels, and 0.9 can classify 3 or 4 levels [27]. Results indicate that the CDS could help differentiate 3 or 4 strata levels of the caregiving difficulty of the mothers of children with CP, which is an excellent result [38]. CDS had a high item separation index.

Meanwhile, the classic method of scale development has been verified through factor analysis. Park [20] verified the validity of CDS in mothers of children with CP. However, scales verified by factor analysis are sometimes adapted and used in other cultures, and sometimes used for groups with different personalities from the target group to which they responded during the scale development. Therefore, to evaluate the fit and difficulty of the items accurately even for the items whose validity has been confirmed through confirmatory factor analysis, it is necessary to verify them with other statistical methods that are less affected by the respondent’s characteristics [39]. This study identified additional accurate psychometric properties of CDS such as the fit and difficulty of items as compared to Park’s [20] previous confirmatory factor analysis results.

Since most of the measurement tools that have been used so far consist of an ordinal scale, the total score does not reflect the level of function implied by each item. In the case of an evaluation tool that uses a total score, an interval scale is a prerequisite for evaluation, as it allows order comparison; however, the differences between individuals cannot be compared with the evaluation based on the ranking scale. The Rasch analysis used a logit score, wherein an ordinal scale is converted into an interval scale so that the order and difference can be directly compared to the ability of the participant and the difficulty of the item [37]. In this study, the measurement results using CDS were composed of ordinal scales that were converted into interval scales using Rasch analysis and analyzed to derive the validated results. This study is meaningful in that it confirmed the psychometric characteristics of the CDS through a Rasch analysis, which was previously only validated through factor analysis.

A limitation of this study is that it did not provide information on the children’s concomitant disabilities. One of the factors that can affect the burden of support for mothers of children with CP is the type and extent of concomitant disorders. As this study investigated the psychometric characteristics of the tool through Rasch analysis, which is not affected by the subject’s characteristics, the presence or absence of concomitant disorders did not affect the results. However, in future studies, it will be necessary to investigate the effect of concomitant disability and the degree of disability on the burden of support in children with CP. Another limitation of this study was related to the re-test reliability and inter-rater reliability. Further studies should be performed to verify the re-test and inter-rater reliabilities of CDS. In addition, in future studies, it is considered necessary to compare CDS with other tools that are used for adaptive functions, in order to confirm the concurrent validity.

## Conclusions

This study employed Rasch analysis to investigate the psychometric characteristics of the CDS as a tool to measure the burden of care for children with CP in 206 mothers. There were no misfit items among all 25 items, and unidimensionality was verified. As a result of the item difficulty, it was found that item 19 had the highest level of difficulty, and item 3 had the lowest level of difficulty. The difficulty distribution of the questions was similar to the mother’s ability distribution, indicating that the difficulty of the questions was appropriate. The person separation index of 2.79 and the item separation index of 3.06 was an acceptable level. The results of the rating scale analysis of the CDS suggest that the Korean version of the CDS could help evaluate the burden of care for children with CP. Future studies should consider investigating various psychometric characteristics such as inter-rater reliability and retest reliability to examine the usefulness of the CDS.

## Data Availability

The data available from the corresponding author on reasonable request.

## Abbreviations

CDS: Caregiving Difficulty Scale
CP: cerebral palsy
GMFCS: Gross Motor Function Classification System
K-CDS: Korean version of the Caregiving Difficulty Scale
MNSQ: mean square
